# A Case of General Anaesthesia Using an I-gel Airway for MRI of an Adult Patient With Athetoid Cerebral Palsy

**DOI:** 10.7759/cureus.35631

**Published:** 2023-03-01

**Authors:** Kenichi Takechi, Aki Yamashita, Ichiro Shimizu

**Affiliations:** 1 Department of Anaesthesia, Matsuyama Red Cross Hospital, Ehime, JPN

**Keywords:** i-gel airway, athetoid cerebral palsy, supraglottic airway device, anaesthesia outside of the operating room, general anaesthesia for mri

## Abstract

Patients with athetoid cerebral palsy may develop cervical myelopathy owing to repetitive involuntary motion. In these patients, MRI evaluation is required; involuntary motion is problematic, and general anaesthesia and immobilisation may be necessary. However, MRI studies requiring muscle relaxation and general anesthesia in adults are rare.

A 65-year-old man with a history of athetoid cerebral palsy required an MRI of the cervical spine under general anaesthesia. General anaesthesia was administered with 5 mg of midazolam and 50 mg of rocuronium in a room adjacent to the MRI room. The airway was secured using an i-gel airway, and the patient was ventilated using a Jackson-Rees circuit. As the only MRI-compatible monitoring method available at our institution was SpO_2_ monitoring, blood pressure was monitored by palpation of the dorsal pedal artery, and ventilation was monitored visually by an anaesthesiologist in the MRI room. The MRI was uneventful. After scanning, the patient awoke promptly and was returned to the ward.

An MRI scan under general anaesthesia requires monitoring of the patient, securing of the airway and ventilation, and careful selection of suitable anaesthetic agents. Although MRI scans requiring general anaesthesia are rare, anaesthesiologists should be prepared for this eventuality.

## Introduction

Patients with athetoid cerebral palsy may develop cervical myelopathy due to repetitive involuntary motion [1]. In these patients, evaluation requires magnetic resonance imaging (MRI); however, poor imaging due to involuntary motion is problematic, and general anaesthesia and immobilisation may be required. However, MRI studies requiring muscle relaxation and general anesthesia in adults are rare.

An MRI under general anaesthesia requires monitoring of the patient, securing of the airway and ventilation, and careful selection of anaesthetic agents [2]. However, there are few detailed reports on general anaesthesia and immobilisation during MRI scans of an adult patient. We report the case of a 65-year-old man with a history of athetoid cerebral palsy who required an MRI of the cervical spine under general anaesthesia.

This manuscript adheres to applicable EQUATOR guidelines. The patient provided written informed consent for the publication of this case report.

## Case presentation

A 65-year-old man (158 cm, 47 kg) with a history of athetoid cerebral palsy was ambulatory and independent in activities of daily living until two weeks prior to examination. The patient had a history of hypertension controlled with medication and laboratory findings were within normal limits. The patient developed sensorimotor deficits in the upper and lower extremities and was unable to walk. MRI evaluation of the cervical spine was attempted; however, the image quality was poor because of involuntary head movements. The anaesthesiologist requested sedation and immobilisation during the MRI scan. We obtained the patient's consent for the MRI scan to be conducted under sedation or general anaesthesia.

The patient was prepared in a room adjacent to the MRI room (MRI zone II). Electrocardiography (ECG), non-invasive blood pressure (NIBP), and oxygen saturation (SpO_2_) monitoring were performed, and oxygen was administered at 10 l/min via a face mask. Our facility did not have an MRI-compatible anaesthesia workstation and syringe pump, and the only MRI-compatible monitoring was an SpO_2_ monitor. Therefore, we first attempted monitored anaesthesia care. Midazolam was administered slowly in divided doses of 1 mg for a total of 3 mg. Five minutes later, the patient was unresponsive to call, the eyelash reflex had disappeared, and spontaneous breathing was maintained. However, he continued to have involuntary head movements that were inappropriate for MRI.

**Figure 1:**
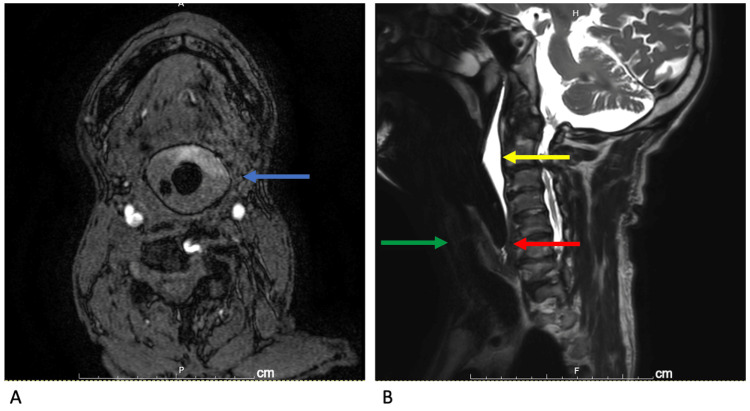
MRI A: MRI angiography horizontal view. Blue arrow: The airway is sealed completely by the i-gel. B: MRI T2-weighted sagittal view. The image shows good separation of saliva from the airway. Green arrow: glottis. Yellow arrow: saliva. Red arrow: tip of i-gel.

The decision was made to switch to general anaesthesia. Oxygen was administered using a face mask and disposable Jackson-Rees circuit containing no metal parts. After the administration of additional midazolam (2 mg) and rocuronium (50 mg), an i-gel #4 device (Intersurgical Ltd, Wokingham, UK) was inserted to secure the airway. The insertion of the i-gel device was smooth, and ventilation was good with negligible leakage. After 10 min of observation in the side room, the patient was moved to the MRI room (MRI zone IV) with continued ventilation using the Jackson-Rees circuit. As the only available MRI-compatible monitoring method available at our institution was SpO_2_ monitoring, blood pressure was monitored by palpation of the dorsal pedal artery, and ventilation was monitored visually in the chest by an anaesthesiologist who ventilated the patient in the MRI room. The MRI took 35 min, but no events occurred that would warrant consideration of poor ventilation or additional medications. The MRI images were of excellent quality (Figure 1). MRI confirmed cervical myelopathy at the C2-6 level and a diagnosis of paralysis due to cervical myelopathy was made.

The patient was moved to the side room after the completion of the scan. He was reattached for ECG, NIBP, and SpO_2_ monitoring. Flumazenil (0.5 mg) and sugammadex (200 mg) were then administered. The patient awoke promptly, and the i-gel was removed. The patient had no memory of the i-gel insertion or MRI scan. After 10 min of observation, he was returned to the ward.

## Discussion

Adult patients with athetoid cerebral palsy can develop cervical myelopathy due to repeated involuntary motion. In the present case, paralysis was caused by an acute exacerbation of cervical myelopathy. Cervical rest is required for cervical myelopathy, but it is sometimes difficult to maintain cervical spine stability with a Philadelphia collar in cases of involuntary motion such as in this patient. The patient suffered frequent involuntary motion of the head and complained of difficulty in tolerating the prolonged MRI scan; therefore, we decided to use general anaesthesia during the MRI.

Guidelines state that general anaesthesia requires minimum monitoring of ECG, SpO_2_, NIBP, and capnography, which should be initiated before anaesthesia induction and continued during anaesthesia [3]. The only method compatible with MRI scans at our institution is SpO2 monitoring, and as in previous MRI cases, it was possible to preserve spontaneous respiration and conduct the scans under light sedation. MRI-compatible monitoring equipment is very expensive. Measures to be taken for patients requiring MRI under general anaesthesia should be discussed and prepared in advance among the relevant departments at each facility [2].

Appropriate airway management during MRI scans has been discussed in several papers [4-6]. The tracheal tube and supraglottic airway may contain metal pilot balloon components that could affect MRI findings [4]. One study reported that the use of a supraglottic airway during an MRI scan under general anaesthesia or deep sedation significantly improves image quality [5]. A previous study compared the i-gel to the LMA-supreme (LMA-supreme, The Laryngeal Mask Company Ltd, St Helier, Jersey, UK) using MRI. The study demonstrated that the i-gel airway allows greater dilation of the upper oesophageal sphincter, while the LMA-supreme compresses the laryngeal inlet [6]. These airway devices had similar insertion success and clinical performance in a simulated difficult airway situation [7]. The manufacturer’s instructions for LMA-supreme mention an associated temperature rise during MRIs, but the i-gel instructions do not. There are case series that have shown i-gel to be useful during MRI scans of adult patients [8]. MRI in this case showed good airway sealing with the i-gel and good separation of saliva and other secretions from the airway. There were no problems with compression of the cervical spine (Figure 1). The i-gel is therefore considered suitable for securing the airway during an MRI.

Regarding MRI with general anaesthesia in adults, a case series using continuous intravenous propofol has been reported [8]. MRI-compatible syringe pumps are expensive and not available at our institution. In addition, we did not use a combination of a long extension tube and syringe pump for continuous administration from outside the MRI room because while using these methods, the door of the MRI room could not be closed. This causes several problems with the infusion line and pump alarms [9]. We chose midazolam as the sedative. The advantages of midazolam over other sedatives during MRI include less circulatory depression, benzodiazepine-associated anterograde amnesia, ease of use in bolus administration, and antagonism with flumazenil. We selected rocuronium as the muscle relaxant. The advantages of rocuronium over other muscle relaxants include its rapid onset of effect and ability to be antagonised by sugammadex.

## Conclusions

In conclusion, we report the anaesthetic management of a patient with cervical myelopathy associated with athetoid cerebral palsy who required general anaesthesia for an MRI scan. Although MRIs requiring general anaesthesia in adults are rare, anaesthesiologists should be aware of the equipment available at the institution and prepare for these eventualities.
